# Do more pregnancies increase the risk of periodontal disease?

**DOI:** 10.12688/f1000research.155151.2

**Published:** 2025-02-06

**Authors:** Mohammad Helmi, Eman AlJoghaiman

**Affiliations:** 1 Department of Periodontics and Community Dentistry, King Saud University, Riyadh, Riyadh Province, Saudi Arabia; 2 Department of Preventive Dental Science, College of Dentistry, Imam Abdulrahman Bin Faisal University, Dammam, Eastern Province, Saudi Arabia

**Keywords:** Parity; Multiple Pregnancy; Periodontal Disease; NHANES

## Abstract

**Background:**

Hormonal changes in pregnancy and their induced effect on periodontal health are well documented. The present study is aimed at the potential repercussions of multiple pregnancies on periodontal health.

**Materials and methods:**

Our study utilized data from key sections of the NHANES. All the pertaining and relevant data for the study is collected. Our exposure variable was the number of pregnancies, and the outcome variable was periodontal disease. The number of pregnancies is classified as one, two, three, four, or more. Age, gender, race/ethnicity, education, poverty/income ratio, marital status, and other variables. Multiple logistic regression models were employed to assess the impact of multiple pregnancies on periodontal disease.

**Result:**

The crude and multiple logistic regression analyses revealed that none of the variables were significantly associated with the prevalence of periodontitis. In univariate analysis, patients with one or two pregnancies had higher odds of experiencing periodontitis (OR 1.154, 95% CI 0.748-1.779), (OR 1.464, 95% CI 0.864-2.483) respectively. However, these associations did not reach statistical significance.

**Conclusion:**

Within the limitation of the study, there is no significant relationship between parity and the prevalence of periodontitis, the longitudinal study may be warranted to delve deeper into any potential associations.

## Introduction

Periodontal diseases are influenced by a variety of factors. ^1^ This prevalent oral condition is initiated by the accumulation of dental biofilm and is further exacerbated by various local and systemic elements. ^2^ Notably, hormonal factors play a significant role in impacting periodontal health. ^3^ Fluctuations in progesterone and estrogen levels during different life stages, including puberty, pregnancy, and menopause, have been identified as contributors to adverse effects on periodontal health. ^4^ The hormonal influence leads to gingival changes that worsen pre-existing dental biofilm-induced gingivitis. Moreover, the absence of estrogen, without the presence of dental biofilm, can result in desquamative changes in the gingiva, representing the other end of the hormonal spectrum. ^5^

During the transitional phases between puberty and menopause, the nine-month duration of pregnancy introduces alterations to periodontal health, manifesting as both localized and generalized changes in the gingiva. Localized changes are characterized by the presence of a pregnancy tumor, while generalized changes manifest as an overall enlargement of the gingiva. ^6^ This period is associated with heightened inflammation of the gingival tissues, commonly known as pregnancy gingivitis. Symptoms include redness, tenderness, and swelling of the gingiva, accompanied by spontaneous bleeding or bleeding during routine activities such as tooth brushing or eating. ^7^ Typically commencing in the second month of pregnancy, these changes can peak in severity during the third trimester. ^8^

The pathogenesis of altered periodontal health during pregnancy involves a combination of factors. ^4^ Studying multiple pregnancies, as opposed to single pregnancies, is critical because cumulative gestational exposure to hormonal fluctuations and immune adaptations may exacerbate oral inflammatory responses, potentially amplifying periodontal vulnerability over successive pregnancies. A key aspect is the rise in levels of hormones such as oestradiol, oestriol, and notably, progesterone. These hormones play a central role in modifying the host immune-inflammatory response to oral bacteria. Specifically, Prevotella intermedia, a Bacteroides species thriving on estrogen and progesterone, experiences a significant increase during pregnancy, serving as a primary bacterial factor. ^9^

Furthermore, these hormones exhibit specific receptors on gingival fibroblasts and epithelial cells, influencing gingival changes. Additionally, they act on endothelial cells, increasing vascular permeability and contributing to the overall alterations observed in the gingiva during pregnancy. ^10^ This intricate interplay underscores the multifaceted nature of periodontal health dynamics in the context of pregnancy.

Progesterone emerges as the key hormone driving these changes, yet estrogen also plays a significant role in inducing vascular changes. ^11^ Simultaneously, deficiencies in host inflammatory cells, particularly in neutrophil chemotaxis, contribute to the adverse aspects of periodontal health. Significantly, vitamin D deficiency is a prevalent concern in the pregnant population, as highlighted in various publications. ^12^ The role of vitamin D in periodontitis is well-documented.

The susceptibility to oxidative stress increases during pregnancy due to heightened metabolic demands and increased tissue oxygen requirements, a factor strongly implicated in the inflammatory process. ^13^ Additionally, the presence of gestational diabetes may further disrupt host defense mechanisms, altering the delicate balance between health and disease. ^14^ The intricate interplay of these hormonal, nutritional, and metabolic factors underscores the complexity of periodontal health dynamics during pregnancy.

While numerous studies have delved into the connection between pregnancy and periodontal disease, there is a scarcity of research exploring the impact of multiple pregnancies on the increased risk of periodontal issues. ^15^^,^^16^ Given the tissue changes occurring with each pregnancy and the cumulative effect of repeated exposure in subsequent pregnancies, there is a hypothetical expectation of a deteriorating impact on periodontal health. ^4^ Therefore, understanding the potential repercussions of multiple pregnancies on periodontal health holds significant importance for both expectant mothers and healthcare professionals.

By acknowledging this potential correlation, healthcare providers can underscore the importance of maintaining good oral hygiene practices and seeking appropriate dental care during pregnancy to mitigate any possible risks. In this context, an effort has been made to investigate the relationship between multiple pregnancies and periodontal health. This exploration aims to enhance our comprehension of potential risks and preventive measures associated with periodontal disease in individuals experiencing multiple pregnancies. Such knowledge can serve as a guide for healthcare professionals, enabling them to offer pertinent advice, facilitate early detection, and provide timely interventions to support optimal periodontal health outcomes in pregnant women. Existing evidence on parity and periodontal disease derives largely from high-income settings, with limited data from regions like [low-income countries], where higher fertility rates and disparities in oral healthcare access may uniquely shape risk trajectories. This study addresses this gap by examining the relationship in a population with demographics, offering insights critical to tailored prevention strategies.

## Methods

### Study design and population

The National Health and Nutrition Examination Survey (NHANES) is a comprehensive, cross-sectional survey conducted in the United States, aiming to provide a nationally representative overview of non-institutionalized individuals living in households. Participants in this survey undergo a series of assessments, including the completion of a questionnaire, medical and dental examinations, and various laboratory tests. The protocols for collecting oral health data in the NHANES 2011–2012 and NHANES 2013–2014 cycles were approved by the Centres for Disease Control and Prevention National Centre for Health Statistics Research Ethics Review Board. Written informed consent was secured from all survey participants.

Our study utilized data from key sections of the NHANES, including demographic information, examination results, questionnaire responses, and limited access data. The focus was on individuals aged 18 years and older who underwent a dental examination, with exclusion criteria in place to remove edentulous subjects from our analysis, we ensure a thorough and targeted evaluation of oral health within a diverse and nationally representative sample of the U.S. population. In this study, pregnancy exposure is operationalized as completed pregnancies (live births or stillbirths), excluding miscarriages or terminations, to align with parity metrics commonly used in population health research.

### Variable of interest

**Exposure variable**

Number of pregnancies

**Outcome variable**

Periodontal disease

For this investigation, we used the NHANES complete periodontal examination data to calculate periodontal disease indices using Eke et al. definition of periodontal disease ^17^). According to this definition, periodontal disease was classified as follows: Severe periodontitis: ≥2 interproximal sites with loss of attachment (LOA) ≥6 mm (not on the same tooth) and ≥1 interproximal site with probing depth (PD) ≥5 mm; Moderate periodontitis: ≥2 interproximal sites with LOA ≥4 mm (not on same tooth), or ≥2 interproximal sites with PD ≥5 mm (not on same tooth); Mild periodontitis: ≥2 interproximal sites with LOA ≥3 mm, and ≥2 interproximal sites with PD ≥4 mm (not on same tooth) or one site with PD ≥5 mm and finally, no periodontitis group whose has no evidence of mild, moderate, or severe periodontitis. ^18^

**Covariate variable**

To ensure a comprehensive examination and control for any factors that might influence the outcome, our analysis includes a range of covariates. These covariates serve the crucial purpose of minimizing the impact of potential confounders, allowing us to scrutinize the relationship between the exposure and outcome with greater precision. The diverse set of covariates comprises age, gender, race/ethnicity, education, poverty/income ratio, marital status, occupation, alcohol consumption, dental insurance coverage, dental visit frequency, and body mass index (BMI). Age is categorized into six groups: (18-30), (31-40), (41-50), (51-60), and over 60 years. Gender is identified as either female or male. Race and ethnicity are classified as non-Hispanic White, non-Hispanic Black, Mexican American and other Hispanic, and non-Hispanic Asian. Poverty indices are categorized into low, middle, and high. Marital status is delineated as yes or no. Occupation is categorized as working and non-working. Dental visits are categorized as regular and not regular. Dental insurance coverage is classified as yes or no. The number of pregnancies is classified as one, two, three, four, or more. Lastly, education level is categorized as less than high school, high school, and college graduate or above. By meticulously examining and accounting for these covariates, we aim to obtain results that are closer to the true relationship between the exposure and outcome, free from the confounding effects of other variables.

### Statistical analysis

The data were obtained by consolidating demographic, health questionnaire, clinical examination, and limited access data from NHANES (2011–2012) with corresponding files from NHANES (2013–2014). To ensure unbiased point estimates and accurate variance estimation, considering the complex sampling design of NHANES, we applied proper sampling weights and utilized a licensed version of SAS survey procedures, following the recommendations of the National Centre for Health Statistics and the Centres for Disease Control and Prevention.

An analysis of the demographics and disease status of the study population was conducted using the Rao-Scott chi-squared test. Additionally, both simple and multiple logistic regression models were employed to assess the impact of multiple pregnancies on periodontal disease. The multiple regression model included age, sex, race, income, and education level as explanatory variables. The selection of these potential confounders was based on either current literature evidence or their association with insurance and dental care utilization variables observed in bivariate analysis. In SPSS, discrete missing values were assigned by designating '999' in the primary text box while leaving subsequent fields blank; once applied, this value was labeled to explicitly identify missing data entries in the dataset. The significance level was set at p ≤ 0.05.

## Results

### Summary of the results

Table 1 presents the demographic characteristics of the study subjects, including weighted percentages. Among the 2128 subjects, more than one-quarter were aged over 60 years [insert specific age], 42.9% identified as non-Hispanic white, 34.6% had a high household level, and over half had either an associate or college degree. The majority of subjects had some form of health insurance and had visited the dentist within the 12 months preceding the survey.

**Table 1.** Demographic characteristics of the study subjects (n=2128).

	Total (n)	Percentage % ^a^
**Age**		
18-30 years	482	22.7
31-40 years	327	15.4
41-50 years	347	16.3
51-60 years	357	16.8
More than 60 years	615	28.9
**Race/ethnicity**		
Mexican American	296	13.9
Other Hispanic	181	8.5
Non-Hispanic White	913	42.9
Non-Hispanic Black	426	20.0
Other Races Including Multi-Racial	312	14.7
**Household income**		
Below Poverty Line	644	30.3
Near Poverty	181	8.5
Low-Income	324	15.2
Middle-Income	242	11.4
High-Income	737	34.6
**Education level**		
Less than High School	408	19.2
High School Level	450	21.1
AA or College Degree	1157	54.4
**Weight status (Based on BMI)**		
Underweight	595	28.0
Normal	564	26.5
Overweight	459	21.6
Obese	510	24.0
**Alcohol consumption status**		
Non-drinker	1363	64.1
Drinker	765	35.9
**Diabetes**		
Present	177	8.3
Not present	1951	91.7
**Periodontal disease**		
Severe	2	0.1
Moderate	45	2.1
Mild	172	8.1
Not present	1909	89.7
**Time of most recent dental visit**		
Less than 1 year	1333	62.6
1-2 years	388	18.2
More than 2 years	407	19.1
**Insurance coverage**		
Yes	1500	81.53
No	516	18.47
**Pregnancy**		
Not pregnant	827	38.9
One	161	7.6
Two	323	15.2
Three	295	13.9
Four	229	10.8
More than 4	293	13.8

^a^ Weighted row percentages.

### Effect of sociodemographic factors on the prevalence of periodontitis

The subjects were categorized based on the severity of periodontitis, dividing them into groups of no, mild, moderate, and severe periodontitis (refer to Tables 2 and 3). The prevalence of periodontitis exhibited a significant difference primarily based on the subjects’ age. Subjects in older age brackets were consistently more likely to have some form of periodontitis compared to their younger counterparts. Additionally, the prevalence of periodontitis on average was higher among pregnant subjects compared to those who were not pregnant.

**Table 2.** Subject demographics and demographic predictors of periodontics in study subjects (n=2128).

	Total (n)	No periodontitis %	Mild periodontitis %	Moderate periodontitis %	Severe periodontitis %	*P*-value
**Age**						
18-30	482	91.3%	0.4%	1.5%	6.8%	0.043 *
31-40	327	90.5%	0.0%	1.8%	7.6%	
41-50	347	90.8%	0.0%	2.0%	7.2%	
51-60	357	86.0%	0.0%	1.7%	12.3%	
More than 60 years	615	89.6%	0.0%	3.1%	7.3%	
**Race/ethnicity**						
Mexican American	296	90.5%	0.0%	2.7%	6.8%	0.835
Other Hispanic	181	89.0%	0.6%	2.2%	8.3%	
Non-Hispanic White	913	89.5%	0.1%	2.1%	8.3%	
Non-Hispanic Black	426	89.0%	0.0%	2.1%	8.9%	
Other Races Including Multi-Racial	312	91.0%	0.0%	1.6%	7.4%	
**Household income**						
Below Poverty Line	644	87.9%	0.2%	2.0%	9.9%	0.830
Near Poverty	181	90.1%	0.0%	2.2%	7.7%	
Low-Income	324	91.4%	0.0%	2.5%	6.2%	
Middle-Income	242	89.7%	0.0%	2.9%	7.4%	
High-Income	737	90.5%	0.1%	1.8%	7.6%	
**Education level**						
Less than High School	408	90.9%	0.2%	1.7%	7.1%	0.842
High School Level	450	89.3%	0.0%	1.8%	8.9%	
AA or College Degree	1157	89.2%	0.1%	2.5%	8.2%	
**Pregnancy**						
Not Pregnant	827	90.7%	0.0%	1.5%	7.9%	0.392
One	161	87.0%	0.0%	3.1%	9.9%	
Two	323	89.5%	0.3%	1.9%	8.4%	
Three	295	90.8%	0.3%	2.0%	6.8%	
Four	229	88.6%	0.0%	4.4%	7.0%	
More than 4	293	88.4%	0.0%	2.0%	9.6%	

^a^ Weighted row percentages.

* Statistically Significant at 0.05.

**Table 3.** Subject characteristics and health-related predictors of periodontitis among study subjects (n=2128).

	Total (n)	No periodontitis %	Mild periodontitis %	Moderate periodontitis %	Severe periodontitis %	*P*-value
**Alcohol consumption status**						
Non-drinker	1363	88.9%	0.1%	2.1%	9.0%	0.258
Drinker	765	91.1%	0.1%	2.2%	6.5%	
**Weight status (Based on BMI)**						
Underweight	595	88.6%	.2%	1.7%	9.6%	0.358
Normal	564	88.5%	0.0%	2.3%	9.2%	
Overweight	459	90.8%	0.0%	2.8%	6.3%	
Obese	510	91.4%	0.2%	1.8%	6.7%	
**Diabetes**						
Present	177	87.0%	0.0%	3.4%	9.6%	0.498
Not present	1951	90.0%	0.1%	2.0%	7.9%	
**Education level**						
Less than High School	408	90.9%	0.2%	1.7%	7.1%	0.842
High School Level	450	89.3%	0.0%	1.8%	8.9%	

^a^ Weighted row percentages.

### Effect of health insurance, dental care utilization, and periodontal disease status on the prevalence of periodontics among study subjects

In Table 4, approximately 9.4% of subjects with health insurance and 9.2% of subjects without health insurance exhibited some form of periodontitis. Analyzing periodontitis concerning the time elapsed since the last dental visit, we observed that 11% of subjects who had a dental visit within 12 months prior to the survey had periodontitis. For those with a dental visit more than 12 months but less than 24 months prior, 8.5% had periodontitis, and 9.8% of those with their most recent dental visit more than 2 years before the survey had periodontitis.

**Table 4.** Distribution of periodontitis per dental visits, insurance coverage, occupation, and, marital status among study subjects.

	Total (n=2128)	No periodontitis %	Mild periodontitis %	Moderate periodontitis %	Severe periodontitis %	*P*-value
**Time of most recent dental visit**						
Less than 1 year	1333	89.0%	0.2%	2.4%	8.4%	0.354
1-2 years	388	91.5%	0.0%	2.3%	6.2%	
More than 2 years	407	90.2%	0.0%	1.0%	8.8%	
**Insurance**						
Yes	1500	90.5%	0.0%	1.8%	7.6%	0.532
No	516	90.8%	0.0%	2.0%	7.2%	
**Occupation**						
Working	1134	90.2%	.1%	1.9%	7.8%	0.793
Not Working	994	89.1%	.1%	2.4%	8.4%	
**Marital Status**						
Married	1495	89.7%	0.0%	2.4%	7.9%	0.169
Not Married	520	89.2%	0.4%	1.5%	8.8%	

^a^ Weighted row percentages.

Both crude and multiple logistic regression analyses revealed that none of the variables were significantly associated with the prevalence of periodontitis. In univariate analysis, patients with one and two pregnancies had higher odds of experiencing periodontitis (OR 1.46, 95% CI 0.864-2.483), (OR 1.15, 95% CI 0.748-1.779) respectively. However, these associations did not reach statistical significance (p > 0.05). Patients with dental visits in the 1-2 year range had greater odds (OR 1.13, 95% CI 0.772-1.651) of having periodontitis, but this association was not statistically significant (p > 0.05) (see Table 5).

**Table 5.** Adjusted and Unadjusted Odds Ratio using Logistics Regression Analysis.

Variable	Unadjusted OR (95% CI)	Adjusted OR (95% CI)
**Number of pregnancies**		
Not Pregnant	Ref	Ref
One	0.78(0.51-1.199)	1.46(0.864-2.483)
Two	1.14(0.639-2.044)	1.15(0.748-1.779)
Three	0.90(0.541-1.484)	0.95(0.588-1.521)
Four	0.78(0.45-1.308)	1.08(0.652-1.78)
More than 4	0.98(0.567-1.679)	1.22(0.788-1.897)
**Dental Visit**		
Less than one year	Ref	Ref
1-2 years	0.76(0.509-1.123)	1.13(0.772-1.651)
More than 2 years	0.89(0.613-1.281)	0.91(0.558-1.489)
Insurance		
No	Ref	Ref
Yes	1.23(0.871-1.733)	1.21(0.859-1.713)

No Periodontitis=0 vs. Periodontitis (all three)=1.

## Discussion

The primary aim of this study is to investigate the association between multiple pregnancies and the severity of periodontal disease/periodontitis. According to the study results, there was no discernible difference in the prevalence of periodontitis between individuals with single pregnancies and those with multiple pregnancies. However, it was noted that the prevalence of periodontitis was higher in pregnant individuals compared to non-pregnant ones. The study findings also indicated that patients with two pregnancies had higher odds of experiencing periodontitis than those with only one pregnancy, although this difference did not reach statistical significance. Both unadjusted and adjusted odds ratios for the number of pregnancies suggested higher odds of periodontitis during pregnancy.

The findings of the current study align with earlier reported research. ^19^^,^^20^ Previous studies indicated higher gingival index and periodontal probing depth among women with prior pregnancies compared to primigravida. However, after adjusting for factors such as age, socio-economic status, education, and other associated risk factors, no correlation was identified. Nevertheless, some studies have demonstrated a significant association between increased gingival scores and periodontal probing depth in women with multiple pregnancies compared to those with a single pregnancy. ^21^ Additionally, research has shown heightened gingival inflammation and increased periodontal probing depth during pregnancy. ^22^^–^^24^

Piscoya et al. conducted a study exploring various factors, including the number of pregnancies and the prevalence of periodontitis. Their findings, organized in a hierarchy, revealed that schooling, family income, body mass index, and bacterial plaque were associated with the prevalence of periodontitis, but not with multiple pregnancies. ^20^ Another study concluded that, in addition to other factors, pregnant women with two or more previous births [multigravidae] exhibited significantly higher gingival index and periodontal probing depth scores compared to those with one previous birth. However, the increased gingival index and periodontal probing depth in multigravidae might be attributed to untreated gingival or periodontal disease from the first pregnancy persisting during subsequent pregnancies. Furthermore, factors such as low socio-economic status and lower educational levels could contribute to negligence of oral hygiene, leading to an increased prevalence of periodontitis. ^21^

The variations in study findings may be attributed to several factors. Multiparous women typically tend to be older than prima gravida women, leading to a longer cumulative exposure to etiological agents of disease. Additionally, the absence of treatment during or between pregnancies results in untreated periodontal disease persisting into subsequent pregnancies. ^21^ Multiparous women, especially those with young children, may prioritize other systemic health conditions, diverting attention, time, energy, and finances away from personal dental care. This tendency can result in neglected oral care, increased plaque accumulation, and a higher prevalence of gingival conditions. ^19^

Therefore, the observed higher gingival index and probing depth in multiparous women may be more related to sociodemographic backgrounds than to a true association with parity. It is widely recognized that existing periodontal disease is exacerbated during pregnancy, and pregnancy itself does not directly cause gingival or periodontal disease. Pregnancy-associated physiological changes can superimpose gingival inflammation on pre-existing dental plaque accumulation. If oral health is well-maintained with the absence of gingival inflammation before pregnancy, the condition of pregnancy itself may not induce gingivitis or periodontitis. Notably, existing studies have not taken into account pre-pregnancy gingival inflammation and treatment for the periodontal condition, which could influence the study outcomes. ^20^

Hormonal alterations during pregnancy contribute to an increase in specific periodontal pathogens, such as Prevotella intermedia, which utilizes elevated hormone levels as a nutrient. Physiological microvascular changes observed in pregnancy, coupled with exposure to altered dental biofilm, may exacerbate pre-existing gingival conditions. The surge in estrogen levels, particularly progesterone, reaches a 20-fold increase, leading to changes in vascular permeability that cause gingival swelling and elevated crevicular fluid levels. ^25^ The heightened production of prostaglandins, in addition to the vascular burden, may intensify gingival inflammation, result in the loss of keratinization of gingival epithelium, and foster fibroblast proliferation. Furthermore, the altered host response is characterized by decreased chemotaxis and phagocytic capacity of neutrophils, along with the down-regulation of IL-6 production. This exposes the gingival tissue to microbial attack, resulting in increased gingival inflammation. ^26^

Gestational diabetes compounds the detrimental effects on host immunity, fostering the proliferation of pathogenic microflora and increasing the risk of periodontal disease. ^25^ Repeated exposure to these events during multiple pregnancies is anticipated to lead to heightened periodontal destruction. However, it has been observed that postpartum, there is a substantial decrease in gingival inflammation, and gingival health is often restored to the pre-pregnancy state. ^27^ Furthermore, the experiences of gingival disease during previous pregnancies may prompt individuals to undergo periodontal treatment, contributing to the restoration of gingival health. ^28^ This could explain the absence of a significant difference in periodontitis among individuals with one pregnancy and those with multiple pregnancies in the present study.

There are several limitations to our study. Notably, the exacerbation of existing periodontal conditions during pregnancy is a well-known phenomenon. Unfortunately, our study lacked data on the status of gingival inflammation before pregnancy, which could have influenced the study outcomes. Additionally, being a retrospective cross-sectional study, our investigation relied on data from a study not specifically designed to address our hypothesis, potentially introducing clinical variations in the disease process. The utilization of dental care after the first pregnancy was not explored, and if a significant number of individuals underwent periodontal treatment after the initial pregnancy and before subsequent pregnancies, it might impact the outcomes. While a standardized protocol was followed for the diagnosis of periodontal disease, there remains a possibility of misclassification, albeit likely to be non-differential. Periodontal status was assessed only at the baseline survey, and changes over follow-up were not considered. It is conceivable that individuals initially free of periodontal disease might develop the condition later, potentially leading to an underestimation of the association for those groups. Another key limitation of using pregnancy as a categorical rather than a continuous variable is the potential loss of nuanced information regarding the incremental effects of each additional pregnancy on periodontal health. The exclusion of edentulous participants may have influenced our study findings, as individuals without teeth are unable to develop periodontitis and may represent a subgroup with a history of severe periodontal disease. To address these limitations, future studies should aim for larger longitudinal prospective designs to validate the findings from this initial study.

While the study did not identify a difference in the prevalence of periodontitis between single and multiple pregnancies, the findings hold significance on two fronts. Firstly, the data were obtained from a substantial sample size, highlighting increased gingival and periodontal changes during pregnancy. This underscores the need to educate all women about these findings, aiming to prevent periodontal changes during pregnancy that may impact their regular daily routines. Secondly, as periodontal disease is deemed a risk factor for pregnancy outcomes, its control assumes prime importance. Addressing and managing periodontal health becomes crucial in optimizing pregnancy outcomes.

## Conclusion

In summary, this study has explored the relationship between parity and the prevalence of periodontitis, revealing no significant association between the prevalence of periodontitis and the number of pregnancies. However, a longitudinal study may be warranted to delve deeper into any potential associations.

## Authors’ contributions

Both Authors contributed equally from the idea to the preparing the draft and both authors reviewed and prepared the final draft of the study.

## Ethics and consent

The protocols for collecting oral health data in the NHANES 2011–2012 and NHANES 2013–2014 cycles received approval from the Centres for Disease Control and Prevention National Centre for Health Statistics Research Ethics Review Board. All survey participants provided written informed consent before publishing their information.

https://www.cdc.gov/nchs/nhanes/irba98.htm

## Data Availability

Figshare: Do more pregnancies increase the risk of periodontal disease?, https://doi.org/10.6084/m9.figshare.25662546 ^29^ The project contains the following underlying data:
• Demographical Characteristics (e.g., gender, income, BMI …), Periodontal Disease status, Pregnancy status, and dental visits. Data are available under the terms of the Creative Commons Attribution 4.0 International license (CC-BY 4.0).
