# Supplementary material for: Do more pregnancies increase the risk of periodontal disease?
Source: F1000Res. 2025 Feb 6;13:1238. Originally published 2024 Oct 16. [Version 2] doi: 10.12688/f1000research.155151.2 (PMC11868742; doi:10.12688/f1000research.155151.2)
Supplement: Peer review history

# Reviewer report: Reviewer response for version 2

**DOI:** 10.5256/f1000research.177715.r364984

**Published:** 2025-03-10

**Authors:** Dr. Talal Shihayb

**Affiliations:** 1 Boston University, Boston, Massachusetts, USA

I had a hard time tracking the changes and suggest an easier way of tracking the changes for reviewers.

Is the work clearly and accurately presented and does it cite the current literature?

Yes

If applicable, is the statistical analysis and its interpretation appropriate?

Partly

Are all the source data underlying the results available to ensure full reproducibility?

Yes

Is the study design appropriate and is the work technically sound?

Partly

Are the conclusions drawn adequately supported by the results?

Partly

Are sufficient details of methods and analysis provided to allow replication by others?

Partly

Reviewer Expertise:

Periodontitis-systemic diseases, oral epidemiology,  advanced methods, meta-research in oral health

I confirm that I have read this submission and believe that I have an appropriate level of expertise to confirm that it is of an acceptable scientific standard.

# Reviewer report: Reviewer response for version 2

**DOI:** 10.5256/f1000research.177715.r368196

**Published:** 2025-02-27

**Authors:** Muhammad Anas

**Affiliations:** 1 Bacha Khan College of Dentistry, Mardan, Pakistan

Title: The Association Between Pregnancy and Periodontitis: A Cross-Sectional Study

 Overall Assessment: This study investigates the relationship between pregnancy and periodontitis, providing valuable insights into the impact of pregnancy on gingival health. While the study has several strengths, including a substantial sample size and standardized protocol for diagnosing periodontal disease, it also has some limitations that affect the interpretation of the results.

 Recommendations:

 1. Future studies should aim for larger longitudinal prospective designs to validate the findings from this initial study.

 2. Consider collecting data on the status of gingival inflammation before pregnancy to better understand the impact of pregnancy on periodontal health.

 3. Explore the utilization of dental care after the first pregnancy and its potential impact on the outcomes.

 4. Consider using more nuanced measures of pregnancy, such as a continuous variable, to capture the incremental effects of each additional pregnancy on periodontal health.

 Conclusion:

 In conclusion, while this study provides valuable insights into the relationship between pregnancy and periodontitis, it has several limitations that affect the interpretation of the results. Future studies should aim to address these limitations and provide more robust evidence on the impact of pregnancy on periodontal health.

Is the work clearly and accurately presented and does it cite the current literature?

Yes

If applicable, is the statistical analysis and its interpretation appropriate?

Yes

Are all the source data underlying the results available to ensure full reproducibility?

Yes

Is the study design appropriate and is the work technically sound?

Yes

Are the conclusions drawn adequately supported by the results?

Yes

Are sufficient details of methods and analysis provided to allow replication by others?

Yes

Reviewer Expertise:

Dentistry, Oral Health, Oral and maxalliofaicial Surgery

I confirm that I have read this submission and believe that I have an appropriate level of expertise to confirm that it is of an acceptable scientific standard.

## References

[1] : Public Health Interventions Targeting Maternal Nutrition and Oral Health: A Narrative Review. *Jordan Journal of Dentistry* .2025; 10.14525/JJD.v2i1.08 10.14525/JJD.v2i1.08

# Reviewer report: Reviewer response for version 2

**DOI:** 10.5256/f1000research.177715.r364985

**Published:** 2025-02-12

**Authors:** Nancy Ajwa

**Affiliations:** 1 College of Dentistry, Riyadh Elem University, Riyadh, Saudi Arabia

Thank you Authors for revising the manuscript.

Is the work clearly and accurately presented and does it cite the current literature?

Yes

If applicable, is the statistical analysis and its interpretation appropriate?

Yes

Are all the source data underlying the results available to ensure full reproducibility?

Yes

Is the study design appropriate and is the work technically sound?

Yes

Are the conclusions drawn adequately supported by the results?

Yes

Are sufficient details of methods and analysis provided to allow replication by others?

Yes

Reviewer Expertise:

Public Health, Periodontology, Orthodontology, Dentistry

I confirm that I have read this submission and believe that I have an appropriate level of expertise to confirm that it is of an acceptable scientific standard.

# Reviewer report: Reviewer response for version 1

**DOI:** 10.5256/f1000research.170283.r343703

**Published:** 2024-12-26

**Authors:** Nancy Ajwa

**Affiliations:** 1 College of Dentistry, Riyadh Elem University, Riyadh, Saudi Arabia

The current structure provides a good foundation for the research paper. I have a few minor comments on the paper, which I am splitting in the headings so that authors can easily identify and improve the respective sections.

**Introduction:**

 -Consider moving some of the detailed descriptions of the hormonal mechanism to later paragraphs for better flow.

 -Add a brief sentence early in the introduction highlighting why studying multiple pregnancies (versus single pregnancy) is particularly important

 -Consider mentioning any geographical or population-specific gaps in current knowledge that this study helps address

**Methods:**

 -Clarify the definition of pregnancy exposure (whether it includes completed pregnancies only or all pregnancies)

 -Add information about how missing data was handled in the analysis

**Results:**

 -Round the odds ratios to 2 decimal places instead of 3 for better readability

 -Include smoking status data in Table 1, as it's an important variable that was mentioned in the methods but not shown in the results

**Discussion:**

 -Add a brief discussion of the limitations of using pregnancy as a categorical rather than continuous variable

 -Include a short paragraph acknowledging the potential impact of excluding edentulous participants on the study findings

 These modifications would improve the manuscript while maintaining its overall scientific merit and indexing. The core findings and conclusions remain sound, and these suggested changes are minor in nature.

Is the work clearly and accurately presented and does it cite the current literature?

Yes

If applicable, is the statistical analysis and its interpretation appropriate?

Yes

Are all the source data underlying the results available to ensure full reproducibility?

Yes

Is the study design appropriate and is the work technically sound?

Yes

Are the conclusions drawn adequately supported by the results?

Yes

Are sufficient details of methods and analysis provided to allow replication by others?

Yes

Reviewer Expertise:

Public Health, Periodontology, Orthodontology, Dentistry

I confirm that I have read this submission and believe that I have an appropriate level of expertise to confirm that it is of an acceptable scientific standard.

# Response

**Published:** 2025-01-30

**Authors:** Eman AlJoghaiman

**Affiliations:** Imam Abdulrahman Bin Faisal University, Dammam, Eastern Province, Saudi Arabia

Dear Reviewer,

 Thank you for your insightful review and valuable suggestions to enhance our manuscript. We have carefully revised the manuscript and highlighted the changes accordingly.

 We sincerely appreciate your time and thoughtful feedback.

 Best regards,

# Reviewer report: Reviewer response for version 1

**DOI:** 10.5256/f1000research.170283.r332865

**Published:** 2024-11-04

**Authors:** Dr. Talal Shihayb

**Affiliations:** 1 Boston University, Boston, Massachusetts, USA

The authors assessed the effects of number of pregnancies on periodontitis. Studying pregnancy outcomes in relation to oral health is crucial. However, I have some comments on the paper:

**Introduction**

 No comments.

**Methods**

 Can the authors explain why they only selected the 2011-2012 and 2013-2014 cycles of NHANES?

 The authors excluded edentulous participants from their study. Dealing with edentulous patients is tricky and not straight forward. If the reason for being edentulous was due to periodontal disease, the perhaps edentulous participants may be considered as the most severe form of periodontitis (since the authors are studying prevalent periodontitis and not incident periodontitis). Given that the authors are studying the accumulated exposure of pregnancy on periodontal disease, the results could be different had edentulous participants due to periodontal disease been included in the analysis, I suggest the authors add edentulous as a separate category or at least justify/discuss excluding edentulous participants from the study in the methods/discussion section.

 If by edentulous participants, the authors meant without any teeth, then any person with 1 tooth is automatically classified as mild periodontitis or no periodontitis and cannot be classified as moderate or severe. However, all included participants should have the chance to be in group of periodontitis, which is unfortunately prevented including participants with just 1 tooth. The authors perhaps may have already included those with at least 2 permanent teeth and if so, should make this clear. If not, then inclusion of participants with at least 2 teeth (if authors are still excluding edentulous participants) in the study or at least discussion of the impact of this aspect on the result is important.

 The authors defined the exposure as the number of pregnancies. Did the authors include all pregnancies whether completed or not? Providing more details on this aspect would make the paper more clear. Furthermore, the authors used it as a categorical variable instead of a continuous exposure. Unfortunately, this results in loss of information. I suggest the authors add this to the discussion section.

 The authors have defined the outcome of chronic periodontitis according to CDC/AAP. I suggest replacing the word periodontal disease with chronic periodontitis in the manuscript as the term periodontal disease is an umbrella term that includes multiple conditions.

 I suggest adding more details on how smoking and alcohol were classified or at least cite their webpages if the authors classified them as NHANES originally did.

 The authors wrote the following under statistical analysis: “To ensure unbiased point estimates and accurate variance estimation, considering the complex sampling design of NHANES, we applied proper sampling weights and utilized a licensed version of SAS survey procedures, following the recommendations of the National Centre for Health Statistics and the Centres for Disease Control and Prevention.” I think using clustering variables to correctly estimate standard errors should be added as both sampling weights and clustering are need to correctly estimate point estimates and standard errors, respectively.

 The authors have pointed out the covariates that they included in their study and mentioned the following under statistical analysis: “The multiple regression model included age, sex, race, income, and education level as explanatory variables. The selection of these potential confounders was based on either current literature evidence or their association with insurance and dental care utilization variables observed in bivariate analysis. The significance level was set at p ≤ 0.05, ensuring a rigorous evaluation of the relationships within the study.” Determining how the confounding variables were selected is very crucial in order to estimate the causal effect of multiple pregnancies on chronic periodontitis. In this study, the authors rightly determined age, sex, race, income, and education level as confounding variables based on previous knowledge and literature. However, the following on insurance and dental visit was not clear: “their association with insurance and dental care utilization variables observed in bivariate analysis.” Furthermore, assessing associations or confounding variables based on p-values should be avoided (please check [1],[2])

As a point related to the one above and based on the criteria the authors went with for determining confounding variables, unfortunately, smoking and diabetes (well-known confounding variables) were left out of the multivariable logistic regression model. Therefore, residual confounding exists in the result. I suggest adding them to the model or at least discuss how the residual confounding of these would impact the odds ratio of number of pregnancies on periodontitis.

**Results**

 The authors did not elaborate on any missing data. Authors should show the frequency and % of missing data for each variables. In addition, authors should describe how they dealt with any missing data in their analysis and discuss its implications on the results.

 The authors mentioned that they have included the following covariates: “age, gender, race/ethnicity, education, poverty/income ratio, marital status, occupation, smoking habits, alcohol consumption, dental insurance coverage, dental visit frequency, and body mass index (BMI)” Although marital, status and occupation were mentioned in table 4, they along with smoking habits were not mentioned in table 1. Smoking habits was not even mentioned in any table. Authors should add these.

 In table 5, patients wrote: “Patients with dental visits in the 1-2 year range had greater odds (OR 1.129, 95% CI 0.772-1.651) of having periodontitis, but this association was not statistically significant (p > 0.05) (see Table 5).” The odds ratios of the confounding variables are of no interest to the authors in the study as they are do not correctly estimate their causal effect. This phenomena is well-known as Table 2 fallacy ( 3). Kindly just report the odds ratios of main variable of interest (number of pregnancies) and remove the other from text or table.

 Please round the odds of pregnancy on periodontitis to 2 digits as 3 digits adds nothing and just complicates reading the results.

 I suggest removing displaying/discussing the results in text as significant or non-significant and instead focus on the point estimates as well as their precision (please check [1]and[2] )

**Discussion and conclusion**

 The authors wrote in the limitations: “Additionally, being a retrospective cross-sectional study, our investigation relied on data from a study not specifically designed to address our hypothesis, potentially introducing clinical variations in the disease process.” The word retrospective should be removed as this was a cross-sectional study.

 Authors should further discuss the impact of no-temporality, pregnancy misclassification likelihood and effect, missing data, and leaving out important confounding variables.

Is the work clearly and accurately presented and does it cite the current literature?

Yes

If applicable, is the statistical analysis and its interpretation appropriate?

Partly

Are all the source data underlying the results available to ensure full reproducibility?

Yes

Is the study design appropriate and is the work technically sound?

Partly

Are the conclusions drawn adequately supported by the results?

Partly

Are sufficient details of methods and analysis provided to allow replication by others?

Partly

Reviewer Expertise:

Periodontitis-systemic diseases, oral epidemiology,  advanced methods, meta-research in oral health

I confirm that I have read this submission and believe that I have an appropriate level of expertise to confirm that it is of an acceptable scientific standard, however I have significant reservations, as outlined above.

## References

[1] : Abandoning statistical significance is both sensible and practical.2019; 10.7287/peerj.preprints.27657v1 10.7287/peerj.preprints.27657v1

[2] : P-value, compatibility, and S-value. *Glob Epidemiol* .2022;4: 10.1016/j.gloepi.2022.100085 100085 10.1016/j.gloepi.2022.100085 37637018 PMC10446114

[3] : A scoping review of Table 2 fallacy in the oral health literature. *Community Dent Oral Epidemiol* .2021;49(2) : 10.1111/cdoe.12617 103-109 10.1111/cdoe.12617 33368566 PMC13244554
